# The Role of Laparoscopic Ultrasonography in the Evaluation of Suspected Choledocholithiasis. A Single-Center Experience

**DOI:** 10.3390/medicina56050246

**Published:** 2020-05-20

**Authors:** Kristaps Atstupens, Maksims Mukans, Haralds Plaudis, Guntars Pupelis

**Affiliations:** 1 Riga East Clinical University Hospital, LV 1038 Riga, Latvia; hplaudis@gmail.com (H.P.); aslimnicagp@gmail.com (G.P.); 2 Statistics Unit, Riga Stradins University, LV 1007 Riga, Latvia; mczedun@gmail.com

**Keywords:** choledocholithiasis, laparoscopic ultrasonography, magnetic resonance cholangio-pancreatography

## Abstract

*Background and objectives:* Opinions differ regarding the optimal diagnostic methods for patients with suspected choledocholithiasis. The aim of this study was to assess the diagnostic accuracy of laparoscopic ultrasonography (LUS) and compare it to pre-operative magnetic resonance cholangio-pancreatography (MRCP); *Materials and Methods:* In all patients with suspected choledocholithiasis LUS was performed during laparoscopic cholecystectomy to evaluate biliary stones. According to availability, part of the patients had pre-operative MRCP. Data for diagnostic accuracy and main outcomes were collected prospectively and analyzed retrospectively; *Results:* Choledocholithiasis was detected in 178 of 297 patients by LUS (59.93%) and in 39 of 87 patients by MRCP (44.8%), *p* = 0.041. LUS yielded a sensitivity of 99.4%, a specificity of 94.3%, a positive predictive value of 96.1% and a negative predictive value of 99.1%. However, pre-operative MRCP had a sensitivity of 61.7%, a specificity of 92.3%, a positive predictive value of 94.9% and a negative predictive value of 51.1%. Moreover, of the 47 patients with no choledocholithiasis by MRCP, in 23 cases it was later detected by LUS (a false negative MRCP finding—38.3%), *p* < 0.001. Median duration of hospitalization was significantly shorter in patients evaluated without pre-operative MRCP—8 days (interquartile range – IQR 11–6) vs. 11 days (IQR 14–9), *p* = 0.001; *Conclusions:* LUS may reduce the role of pre-operative MRCP and can become a rational alternative to MRCP as a primary imaging technique for the detection of choledocholithiasis.

## 1. Introduction

Recently, reported incidence of the common bile duct (CBD) stones varies between 8–20% in patients with gallstone disease [1]. However, concomitant CBD stones are found in 11% to 21% of cases during cholecystectomy [2]. Choledocholithiasis requires a complex approach for the restoration of the biliary drainage function due to the potentially life-threating biliary complications. There is a wide range of diagnostic options which are useful at the peri-operative and intra-operative stage, however, recommendations regarding the timing and method of bile duct imaging vary. MRCP is still recommended as the method of choice at the pre-operative stage [3] followed by therapeutic endoscopic retrograde cholangio-pancreatography (ERCP) in the case of biliary stones as a first step and surgery afterward as a second step. Despite the significant value of MRCP, there are some limitations. MRCP may be less available in high patient flow hospitals, and it has a lower diagnostic accuracy if stones in the bile duct are smaller than 5 mm, especially in the case of biliary pancreatitis. The individual restrictions such as claustrophobia and metallic implants are significant [2].

However, ERCP is an invasive procedure and associated with a 5–10% complication rate (post-ERCP pancreatitis, bleeding, duodenal or bile duct perforation, cholecystitis, etc.). It is associated with a 0.1–1% mortality rate mainly due to papillotomy during the procedure; thereby, ERCP is more suitable for patients with a proven diagnosis and precisely selected indications [4,5]. On the other hand, therapeutic ERCP may be supplemented by endoscopic ultrasonography (EUS), which has high sensitivity and specificity rates—93% and 96%, respectively [2].

Due to the increased use of laparoscopic common bile duct exploration (LCBDE) which is an effective and safe alternative to ERCP, even with some advantages in comparison with the two-step approach [6,7], LUS has been increasingly applied as the primary imaging to screen CBD stones and it is comparable with intraoperative cholangiography (IOC). Despite the significant diagnostic accuracy of IOC (59–100% sensitivity and 93–100% specificity) [2], it is linked to ionizing radiation, increased operative time and a lower diagnostic accuracy than LUS. However, the advantages of LUS include high quality real-time intra-operative diagnostic of choledocholithiasis, no invasiveness, short time for imaging, possibility to repeat imaging at any stage of operation and no ionizing radiation. The aim of the study was an assessment of the diagnostic accuracy of LUS in detecting choledocholithiasis and comparison with pre-operative MRCP.

## 2. Materials and Methods

### 2.1. Patients

The study covers the period from 2012 to 2017; patients who were urgently admitted with signs and symptoms of complicated gallstone disease and a risk of choledocholithiasis were included prospectively [8]. All patients were selected for laparoscopic cholecystectomy and laparoscopic ultrasonography (LUS) to prove or rule out choledocholithiasis. Complicated gallstone disease was suspected based on evidence of acute biliary pancreatitis, acute cholangitis or characteristic biliary colic, frequently accompanied by pale stools, dark urine and jaundice. Pre-operative diagnosis of cholangitis was based on the criteria recommended in the Tokyo Guidelines 2018. The evidence of systemic inflammatory response (increased WBC > 10 × 1000/µL or CRP more than 10 mg/L), cholestatic pattern, presented by abnormal liver function tests (alkaline phosphatase (ALP), gamma glutamyl transferase (GGT), aspartate aminotransferase (AST), alanine aminotransferase (ALT) ≥ 1.5 standard deviation) or bilirubin ≥ 34.2 µmol/L and gallstones in the gallbladder and/or dilation of the common bile duct >6 mm confirmed by trans-abdominal US, were the criteria for inclusion [9,10]. Biliary pancreatitis and cholangitis were diagnosed according to the revised Atlanta 2012 criteria (abdominal pain consistent with acute pancreatitis, serum lipase activity more than three times over the upper limit of normal) and characteristic findings of acute pancreatitis on radiological investigations [11]. The patient’s pre-operative overall physical condition was assessed using the adopted American Society of Anesthesiologists Physical Status classification system (ASA score) [12,13]. Patients with severe acute biliary pancreatitis or cholangitis who were not candidates for laparoscopic surgery and cases with converted operation to open surgery were excluded.

### 2.2. Pre-Operative Diagnosis of Choledocholithiasis

**Figure A1:**
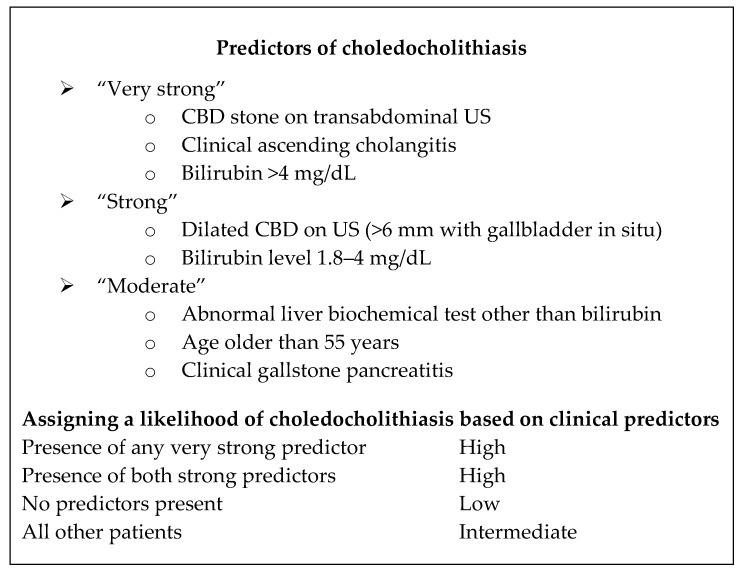
Predictors of choledocholithiasis (American Society of Gastrointestinal Endoscopy) [14].

At the time of admission all patients underwent trans-abdominal ultrasound (TAUS). All TAUS examinations were performed by senior residents supervised by a certified radiologist. Gallstone disease was identified and possible choledocholithiasis or indirect signs of CBD stones, especially the dilation of the CBD more than 6 mm or the dilation of intrahepatic bile ducts were evaluated. According to availability, part of patients had pre-operative MRCP. Risk groups of choledocholithiasis were evaluated according to the ASGE guidelines (Figure A1 in Appendix A) [14].

### 2.3. Laparoscopy and Laparoscopic Ultrasonography

Operations were performed by certified HPB surgeons of our department with a classic four-trocar approach. After the formation of pneumoperitoneum, trocars were positioned in the following way—10 mm through the umbilical ring for the camera and three for the manipulation—one of them 10 mm at the epigastrium proprium and two, 5 mm to the right side. Then the cystic duct was identified and clipped proximally close to the neck of the gallbladder. All LUS were done by a mobile ultrasound machine and a special, flexible probe for laparoscopic ultrasonography.

**Figure 1:**
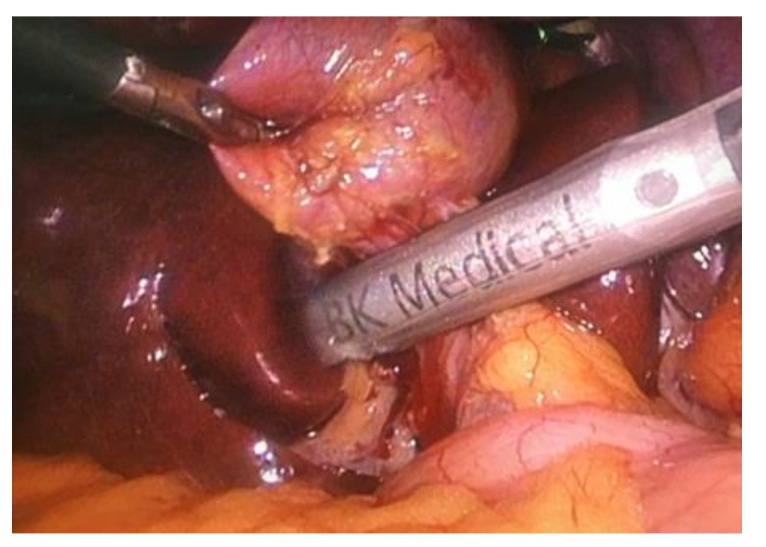
Laparoscopic ultrasonography (LUS) probe on the proximal part of the hepato-duodenal ligament. (photo by the author).

An ultrasound probe was inserted in the abdominal cavity through the epigastric trocar. Once the fundus part of the gallbladder was lifted over the liver, scanning began with a gallbladder examination. The common hepatic duct and common bile duct were scanned when the LUS probe was placed on the superior edge of the hepato-duodenal ligament and slid inferiorly to the distal end of the bile duct (Figure 1).

**Figure 2:**
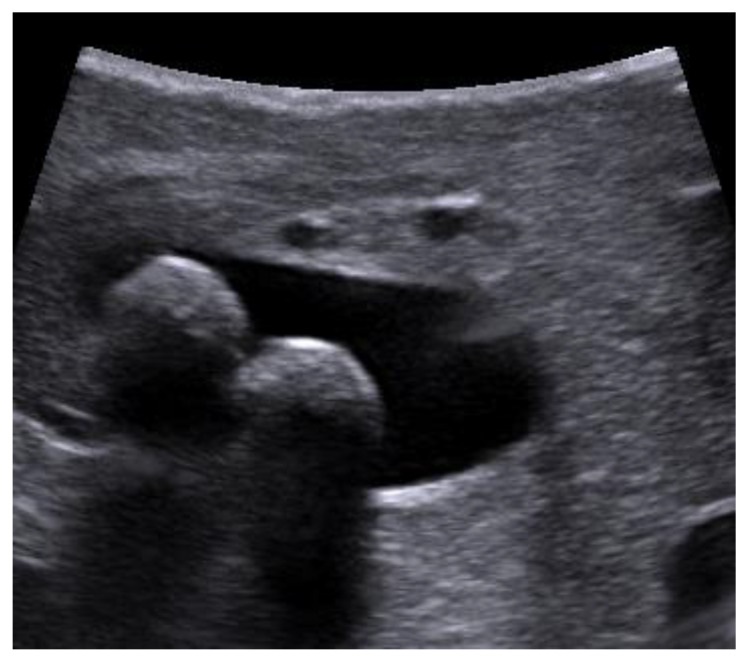
Stones in the bile duct (photo by the author).

The distal part of the common bile duct (retro-duodenal and intra-pancreatic) and the area of the papilla Vateri were scanned through the duodenum. The left and right hepatic ducts and their junction were investigated through the right hepatic lobe. The main metrics examined in the study were the maximum width of the bile duct, its content (gallstones), the maximum size and the quantity of the stones. Stones were considered as a positive finding on LUS or choledocholithiasis (Figure 2), as well as biliary sludge in the lumen of the bile duct.

In case of choledocholithiasis, LCBDE was done following two main approaches—through the cystic duct and directly through the bile duct (ductotomy). The type of the bile duct clearance method was based on the LUS finding (size of the bile duct and stones, the number of stones). However, in cases with sludge in the bile duct, only rinsing without additional bile duct exploration was performed.

In all cases during surgery, biliary drainage function (main purpose) was completely restored. In cases of LCBDE failure or insufficient trans-papillary biliary drainage, choledochostoma (controlled biliary fistula) was left.

All patients with cholangiostomas had a fistulography (cholangiography) on the third day after surgery and only after the re-approval of choledocholithiasis or stenosis of the papilla, ERCP with endoscopic papillotomy was done. All choledochostomas were evacuated at the outpatient stage after the 10th postoperative day.

In cases with an extensive bile duct dilatation and stenosis of the papilla, choledocho-duodenostomies were performed only in patients over 60 years of age.

### 2.4. Statistical Analysis

The interval data were expressed as a median (Me) with an interquartile range (IQR), because the breakdown of the data was asymmetric, confirmed by the Kolmogorov–Smirnov test. The Mann–Whitney *U* test was used to compare the interval data between the groups. Pearson χ2 and Fisher tests were used to compare the nominal data between the groups. The correlation between the pre-operative and total hospital stay and clinical data were calculated by the Spearman rho method. A logical regression analysis was performed to identify factors associated with a longer hospital stay. The results were considered statistically significant at the *p*-value of <0.05 and a confidence interval of 95%. Statistical analysis was performed with the SPSS software (version 20) and MedCalc (version 15).

### 2.5. Ethics

The assessment and usage of all retrospective clinical data were approved and permitted before the study on the 15th of November 2018 by the Ethics Committee of Riga Stradins University (study No. 6–3/56). The study protocol conformed to the ethical guidelines of the “World Medical Association (WMA) Declaration of Helsinki—Ethical Principles for Medical Research Involving Human Subjects” adopted by the 18th WMA General Assembly, Helsinki, Finland, June 1964 and amended by the 59th WMA General Assembly, Seoul, South Korea, October 2008 [15].

## 3. Results

### 3.1. Patient Demographics and ASA Score

According to the criteria, 297 patients were included in the study and underwent a technically successful LUS during laparoscopic cholecystectomy. Patient demographics and ASA scores are available below (Table 1).

### 3.2. Distribution and Characteristics of Patient Groups

In total, 178 cases of choledocholithiasis were diagnosed during LUS, 59.93% (LUS+ group), while in 119 patients or 40.07% choledocholithiasis was not verified (LUS− group).

A significantly higher inflammatory response, higher bilirubin level, more commonly defined mechanical jaundice (according to the Tokyo guidelines 2018) and cholangitis were observed in patients with a positive LUS finding. However, the level of liver enzymes and alkaline phosphatase was similar in both groups. Moreover, significantly higher lipase levels were found in the patient group with a negative LUS finding (LUS−)—a median of 147 (IQR 4323–37), *p* = 0.004. Similarly, biliary pancreatitis was statistically significantly more commonly diagnosed in the LUS− group patients (*p* = 0.005). Acute cholecystitis was equally common in both groups. The most significant laboratory and clinical data are shown in Table 2.

### 3.3. Value of Magnetic Resonance Cholangio-Pancreatography

**Figure 3:**
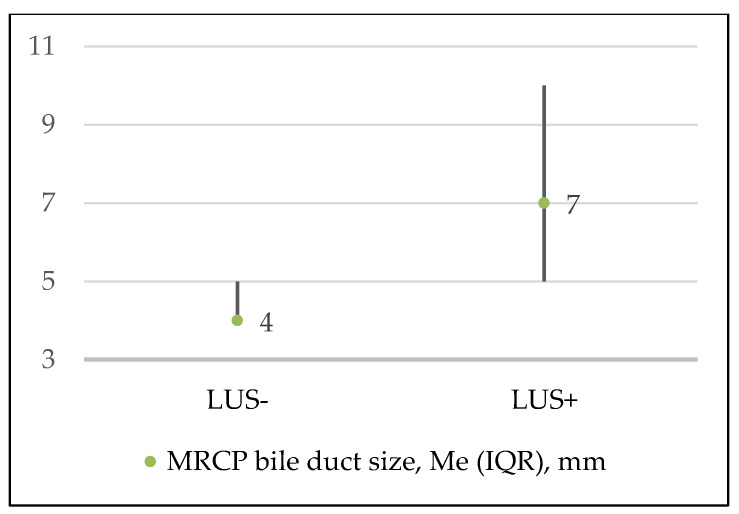
Median values of bile duct sizes on magnetic resonance cholangio-pancreatography (MRCP) in groups.

Preoperative MRCP was performed in 87 patients from the cohort (29.3%). In 23 (26.4%) cases, patients were in the medium risk group and in other 64 (73.6%) cases in the high-risk group of choledocholithiasis (according to the criteria from the ASGE guidelines). There was no statistically significant difference (demographic data, ASA score and clinical data) between the patients with and without preoperative MRCP. The median value (Me) of the bile duct on MRCP in the whole cohort was 6 (IQR 9–4) millimeters and in the LUS+ group it was significantly higher than in the LUS− group, *p* < 0.001 (Figure 3). A moderate positive correlation between these values was observed (r = 0.432; *p* < 0.001).

Overall, in 39 patients (44.8% from all 87 MRCPs) choledocholithiasis was identified at the preoperative stage by MRCP. In 37 of them it was also confirmed during surgery on LUS (a true positive MRCP finding) and in 2 of them it was not confirmed (a false positive MRCP finding). However, out of the other 47 patients with no choledocholithiasis on MRCP, in 23 cases it was detected afterwards during surgery on LUS (a false negative MRCP finding—38.3%), *p* < 0.001. A true negative MRCP finding was observed in 24 patients (Table 3).

Based on the above-mentioned data, the diagnostic value of MRCP was obtained in the study population (Table 4).

A significantly higher diagnostic value of MRCP was observed when patients with sludge and small stones ≤1 mm in the bile duct on LUS were excluded from data analysis (Table 5).

Proven choledocholithiasis on MRCP had a statistically significant correlation with choledocholithiasis during surgery on LUS (r = 0.498; *p* < 0.001). Notably, the size of stones not diagnosed on MRCP (23 patients) was a median of 3 (IQR 3–1) millimeters at the time of surgery diagnosed by LUS; however, the size of the stones diagnosed by both methods (MRCP and LUS) was statistically significantly higher—a median of 5 (IQR 7–4) millimeters, *p* < 0.001.

### 3.4. Value of the Laparoscopic Ultrasonography

**Figure 4:**
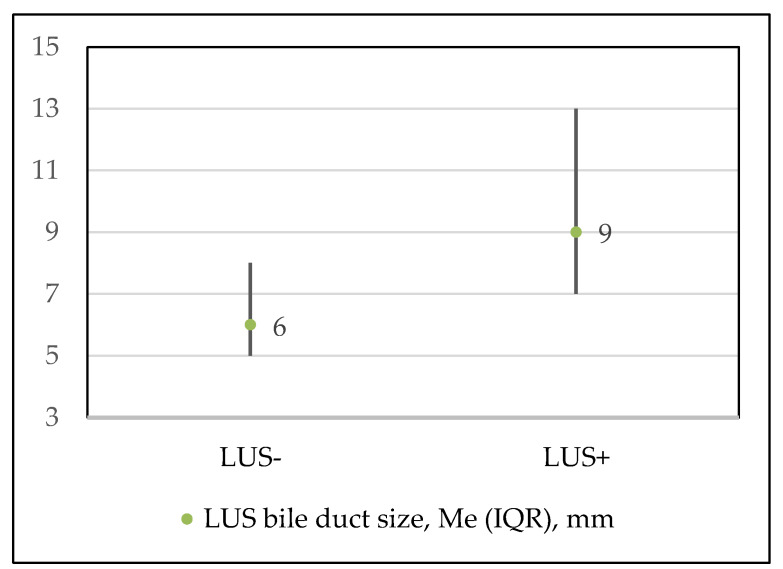
Median values of bile duct sizes on LUS in groups.

LUS was performed in all 297 patients of the study regardless of preoperative imaging and its results. The median size of the bile duct on LUS in the whole cohort was 8 (IQR 11–6) millimeters and it was significantly higher in the LUS+ group patients compared to LUS− (Figure 4), *p* < 0.001. A moderate positive correlation was observed between the bile duct width on LUS and a positive finding (r = 0.511; *p* < 0.001).

**Figure 5:**
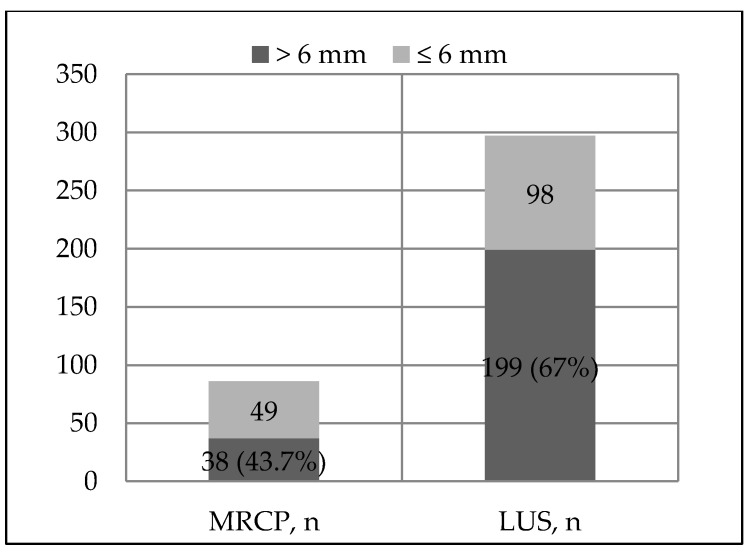
Bile-duct dilatation of cohort patients on MRCP and LUS.

Bile-duct dilatation (>6 mm) in the study population was more commonly found on LUS compared to MRCP (Figure 5).

**Figure 6:**
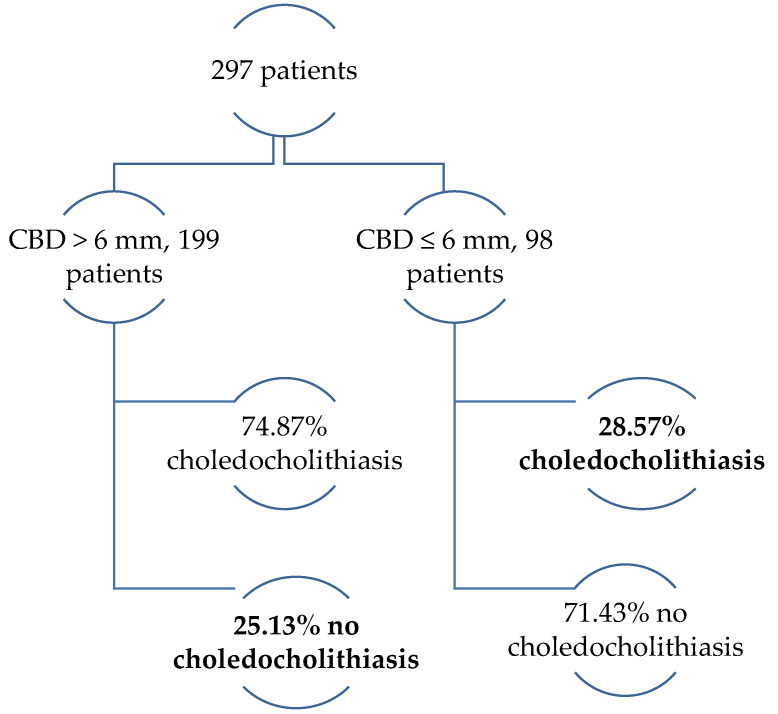
LUS finding depending on bile duct dilatation (>/≤6 mm)**.**

Moreover, binary regression analysis showed a statistically significant (*p* = 0.001) odds ratio of LUS bile duct dilatation in the detection of choledocholithiasis on LUS−8.97 (95% confidence interval 5.0–15.9). During surgery, choledocholithiasis was diagnosed on LUS in 178 patients (59.93%) from the cohort. In 15.8%, the bile duct was not dilated (≤6 mm) and in 84.2% the bile duct was wider than 6 mm. Evidence from the previous studies demonstrated that choledocholithiasis was also frequently observed in a non-dilated (≤6 mm) bile duct recognized on LUS. Contrary to that, in about one quarter of patients with a dilated bile duct (>6 mm) on LUS choledocholithiasis was not found (Figure 6).

During the two-year period, from the cohort of 297 patients a follow-up revealed the necessity to readmit 9 patients (3.13%) due to residual stones, diagnosed previously on LUS in 8 (LUS+ group), while for one patient choledocholithiasis was not diagnosed on LUS during the first intervention (LUS− group). Based on the data, the diagnostic value of laparoscopic ultrasonography was determined (Table 6). All re-hospitalized patients had successfully undergone endoscopic biliary treatment (ERCP).

### 3.5. Duration of Hospitalization

The total hospital stay, and postoperative period were significantly longer in the LUS+ group patients compared to the LUS− group (Table 7).

The following finding is notable as well—the preoperative and total hospital stay was significantly longer in patients in whom preoperative MRCP was done. The postoperative hospital stay did not differ when preoperative MRCP was applied (Table 8).

### 3.6. Complications

The application of LUS was not associated with complications due to the non-invasiveness and the technical feasibility of the method (puncturing or opening of the bile duct and tissue preparation was not necessary), thereby no complications were observed associated with LUS. LUS in all cases was performed successfully without an iatrogenic bile duct lesion or other complications. However, in 5 cases of MRCP motion artefacts were observed leading to an incomplete interpretation of the imaging data. There were no lethal cases in the study group.

## 4. Discussion

Choledocholithiasis is the main complication of gallstone disease and occurs in 11%–21% of patients during cholecystectomy [2]. Recognition of choledocholithiasis requires an accurate and timely investigation due to the potentially serious and fatal complications of the disease. Despite the wide range of diagnostic possibilities, the detection of choledocholithiasis is still a major challenge for surgeons and radiologists. The bile duct stones may be predicted by the criteria of preoperative examination; however, the accuracy is not very high [15,16]. It is important to note that invasive diagnostic methods such as ERCP should not be routinely used in the visualization of choledocholithiasis. Due to the potentially severe complications of ERCP [17] and costs, the indications for this method are very specific and can only be used for therapeutic purposes. MRCP is recommended as the main diagnostic method of complicated gallstone disease in many studies [2]. The rationale for that recommendation is the non-invasiveness of the method, its high diagnostic accuracy and the ability to evaluate the biliary system and pancreas before surgery. On the other hand, some reports indicate an incomplete value of MRCP in patients with small stones in the bile duct. A recently published comparative study also points to the superiority of LUS over MRCP in the diagnosis of choledocholithiasis [18]. Moreover, the availability of the MRCP is limited in many institutions and often is not available for all patients, as it was in our study where it was done in 29.3% of patients. The above-mentioned facts suggest that the optimal diagnostic management of choledocholithiasis is still to be clarified.

The incidence of choledocholithiasis increases with age and the overall risk is higher in elderly patients with severe comorbidities like in our study [19,20,21].

Biochemical markers of the liver (ALT, AST, GGT, ALP) may be useful in predicting incomplete biliary drainage, including due to choledocholithiasis [22], but there is no evidence of changes in these tests in all cases. Insignificant differences between liver transaminases in patients with and without choledocholithiasis were observed in this study. Reports from literature indicate that no specific imaging is required in cases with unchanged liver biochemical parameters and a non-dilated bile duct, because choledocholithiasis is not predictable [16,23]. However, the data of the current study suggest that a significant number of patients may also have stones in a non-dilated bile duct. The direct fraction of bilirubin and the total level of bilirubin were the only statistically significant biochemical indicators that increase in patients with choledocholithiasis. Other authors present similar data, suggesting that only the direct fraction of bilirubin is a very strong predictor of choledocholithiasis, and liver transaminases must be estimated very critically [14].

Many authors suggest MRCP as a first-line diagnostic method of choledocholithiasis [3,24,25,26,27]. In the existing guidelines (ASGE, 2010), MRCP is also recommended for patients with a medium (10%–50%) risk of choledocholithiasis [14,28]. MRCP was used in 23 (26.4%) patients of the medium-risk group in the present study. In other 64 (73.6%) patients who were at high risk for choledocholithiasis according to the criteria from the ASGE guidelines, choledocholithiasis was not found in 34. This finding does not comply with the ASGE recommendations to perform ERCP first in high-risk patients without any specific diagnostic modality before ERCP. A combined preoperative MRCP and LUS applied in the selected patient cohort allowed avoidance of unnecessary ERCP and possible complications related to that. There are similar observations published by other authors [29,30,31].

According to the data from literature, MRCP has a high diagnostic value in the detection of choledocholithiasis [32]; however, there are reports that indicate an insufficient diagnostic value of MRCP in cases with small stones and sludge in the common bile duct [18]. The obtained data are similar—the size (median value) of the undiagnosed stones on preoperative MRCP was 3 mm, complying with the data from literature. However, the sensitivity and specificity of MRCP when the stone size was more than 1 mm reached 82.9% and 92%, respectively. Despite the discussions about the clinical relevance of bile sludge in the common bile duct, some authors have proven the role of sludge in the pathogenesis of biliary pancreatitis [33] and cholangitis [34], as well as a high risk of recidivism of biliary pancreatitis in case of undetected microlithiasis—33%–60%. Microlithiasis verified by LUS is important at the time of surgical intervention. It provides the possibility to flush the bile duct achieving duct clearance. However, there exists another opinion regarding microlithiasis, without any active treatment. This provides that in most of cases biliary sludge may pass through the papilla spontaneously. Several authors also report the significant value of LUS in the detection of sludge compared to other methods [35]. In the current study, in 75.5% of patients choledocholithiasis was diagnosed by LUS considering all high-risk patients, according to the ASGE guidelines. The other 24.5% of patients had no stones in the bile duct. These patients also avoided unnecessary manipulations including ERCP. Unlike MRCP, the indications for possible postoperative ERCP are defined very clearly and precisely during surgery using LUS. If clearance of the bile duct fails, the operation is finished by the formation of cholangiostoma (controlled biliary fistula, mostly trans-cystic for biliary drainage). Postoperative cholangiography with a contrast agent is performed to clarify the condition of the biliary tree and possible evidence of stones for the clarification of the final indications for ERCP thereby minimizing unnecessary invasive procedures. According to the gained experience, the risk of post-ERCP pancreatitis is reduced in patients with cholangiostoma due to the decompression of the biliary tree, the drainage of the bile and pancreatic juice. Other authors describe a similar experience [36].

LUS is one of the newest intraoperative imaging methods of choledocholithiasis; it provides the operating surgeon with very precise information of the bile ducts with a very high diagnostic value. LUS is recommended in the ASGE guidelines in patients with a medium risk of choledocholithiasis (10%–50%) as one of the intraoperative imaging modalities without specific preoperative imaging [14]. Similar to other authors, the diagnostic value of LUS in the current study was very high—99.4% sensitivity and 94.3% specificity. In the present study, the superiority of LUS over MRCP is related to the facts that MRCP may miss stones smaller than <5 mm, especially in patients with biliary pancreatitis [2,37]. In contrast, two cases of MRCP-verified choledocholithiasis were not confirmed by the LUS during laparoscopy. These two false-positive MRCP cases could be explained by the spontaneous trans-papillary migration of the gallstone to the duodenum in the time interval between MRCP and surgery. Other authors share a similar experience [37]. There are reports presented that in up to a third of patients’ spontaneous migration of the gallstones to the duodenum before surgery may occur [38], which is much more than in the present study. Therefore, by using LUS unnecessary LCBDE and complications related to it can be avoided. The diagnostic value of LUS in our study population was also superior to MRCP, similarly to the data presented by other authors [18]. According to our experience, LUS provides the possibility to evaluate the permeability of the papilla Vateri, which was observed during the bile duct rinsing. It was also determined that turbulent movements of the fluid in the lumen of the descendent part of the duodenum can be observed. Good visualization is the cornerstone of surgery. LUS assists the surgeon, and at any time during the operation may quickly, accurately, safely help to control the anatomy so that patients are not exposed to the risk of an iatrogenic lesion of any structures [39,40]. The experience confirms the same; thus, there were no iatrogenic lesions of the bile ducts in patients of the selected study population.

## 5. Conclusions

LUS could be a promising alternative to MRCP as a primary imaging technique for the detection of the choledocholithiasis. LUS allows minimizing expensive and time-consuming examinations in the preoperative period, improving the diagnostic quality, ensures a faster recovery, reduces complications and the duration of hospitalization.

## Figures and Tables

**Table 1:** Demographics and American Society of Anesthesiologists Physical Status classification system (ASA) score of the cohort.

Parameters	Total, *n* (%)	Women, *n* (%)	Men, *n* (%)	*p* Value
Number of patients	297 (100)	210 (70,7)	87 (29.3)	0.001
Age Me (IQR), years	61 (71–46)	58 (68–45)	65 (74–55)	0.035
ASA I	49 (16.7)	42 (20.1)	7 (8)	0.011
ASA II	146 (49.3)	105 (50.2)	42 (48.3)	0.758
ASA III	88 (29.6)	55 (26.3)	32 (36.8)	0.072
ASA IV	14 (4.4)	8 (3.3)	6 (6.9)	0.275

**Table 2:** Biochemical and clinical parameters in groups.

**Biochemical Parameters**	**Cohort Me (IQR)**	**LUS+ Group (*n* = 178)**	**LUS− Group (*n* = 119)**	***p*** **Value**
		**Me (IQR)**	**Me (IQR)**	
CRP, mg/L	7 (24–2)	8.3 (38–4)	6 (15–2)	0.022
ALT, *U*/L	230 (464–94)	243 (473–108)	216 (447–75)	0.155
AST, *U*/L	188 (371–63)	189 (405–58)	188 (333–71)	0.972
ALP, *U*/L	195 (278–130)	216 (316–131)	172 (249–122)	0.061
Bilirubin total, µmol/L	34 (66–15)	39 (84–20)	29 (52–13)	0.001
Bilirubin direct, µmol/L	24 (53–8)	28 (67–10)	18 (37–7)	0.001
Lipase, *U*/L	64 (2268–34)	56.9 (857–32)	147 (4323–37)	0.004
**Clinical Conditions**	**Cohort, *n***	***n*** **(%)**	***n*** **(%)**	***p*** **Value**
Mechanical jaundice	147	100 (56.5)	47 (39.5)	0.009
Cholangitis	134	107 (60.1)	27 (22.7)	<0.001
Cholecystitis	194	118 (66.3)	76 (63.9)	0.813
Biliary pancreatitis	110	55 (30.9)	55 (46.2)	0.005

**Table 3:** Diagnostic value of MRCP in diagnosis of choledocholithiasis, controlled by LUS. * Data available for 86 out of 87 patients.

*n* (%)		MRCP		Total
		−	+	
**LUS**	−	24 (92.1)	2 (7.7)	26 (100)
	+	23 (38.3)	37 (61.7)	60 (100)
**Total**		47 (54.7)	39 (45.3)	86 * (100)

**Table 4:** Diagnostic value of MRCP.

Criteria of Diagnostic Value	%	95% Confidence Interval,%
Sensitivity	61.7	54.6–64.4
Specificity	92.3	76.1–98.6
Positive predictive value	94.9	84.1–99.1
Negative predictive value	51.1	42.1–54.6

**Table 5:** Diagnostic value of MRCP *

Criteria of Diagnostic Value	%	95% Confidence Interval,%
Sensitivity	82.9	73.6–86.9
Specificity	92	76.7–98.5
Positive predictive value	94.4	83.8–99
Negative predictive value	76.7	64–82.1

* Analysis was performed without patients with sludge and stones ≤1 mm on LUS.

**Table 6:** Diagnostic value of LUS.

Criteria of Diagnostic Value	%	95% Confidence Interval,%
Sensitivity	99.4	97.1–100
Specificity	94.3	91–95.1
Positive predictive value	96.1	93.8–96.6
Negative predictive value	99.1	95.7–100

**Table 7:** Duration of hospitalization in groups.

Me (IQR), Days	LUS+ *n* = 178	LUS− *n* = 119	*p* Value
Total hospital stay	10 (13–7)	8 (10–6)	<0.001
Preoperative period	5 (7–3)	5 (7–3)	0.655
Postoperative period	5 (8–3)	3 (4–2)	<0.001

**Table 8:** Duration of hospitalization depending on MRCP.

Me (IQR), Days	MRCP *n* = 87	No MRCP *n* = 210	*p* Value
Total hospital stay	11 (14–9)	8 (11–6)	0.001
Preoperative period	6 (8–5)	4 (6–2)	<0.001
Postoperative period	4 (7–2)	4 (5–2)	0.185

## References

[1] Tozatti J., Mello A.L.P., Frazon O. (2015). Predictor factors for choledocholithiasis. Arq. Bras. Cir. Dig..

[2] Costi R., Gnocchi A., Di Mario F., Sarli L. (2014). Diagnosis and management of choledocholithiasis in the golden age of imaging, endoscopy and laparoscopy. World J. Gastroenterol..

[3] Chen W., Mo J.J., Lin L., Li C.Q., Zhang J.F. (2015). Diagnostic value of magnetic resonance cholangiopancreatography in choledocholithiasis. World J. Gastroenterol..

[4] Maple J.T., Ikenberry S.O., Anderson M.A., Appalaneni V., Decker G.A., Early D., Evans J.A., Fanelli R.D., Fisher D., Fisher L. (2011). The role of endoscopy in the management of choledocholithiasis. Gastrointest. Endosc..

[5] Antonios V., Georgios F., Andreas P. (2015). Endoscopic retrograde cholangiopancreatography-related perforations: Diagnosis and management. World J. Gastrointest. Endosc..

[6] Qiu W., Sun X.D., Wang G.Y., Zhang P., Du X.H., Lv G.Y. (2015). The clinical efficacy of laparoscopy combined with choledochoscopy for cholelithiasis and choledocholithiasis. Eur. Rev. Med. Pharmacol. Sci..

[7] Cuschieri A., Lezoche E., Morino M., Croce E., Lacy A., Toouli J., Faggioni A., Ribeiro V.M., Jakimowicz J., Visa J. (1999). E.A.E.S. multicenter prospective randomized trial comparing two-stage vs single-stage management of patients with gallstone disease and ductal calculi. Surg. Endosc..

[8] Takada T., Strasberg S.M., Solomkin J.S., Pitt H.A., Gomi H., Yoshida M., Mayumi T., Miura F., Gouma D.J., Garden O.J. (2013). TG13: Updated Tokyo Guidelines for the management of acute cholangitis and cholecystitis. J. Hepatobiliary Pancreat. Sci..

[9] Kiriyama S., Takada T., Strasberg S.M., Solomkin J.S., Mayumi T., Pitt H.A., Gouma D.J., Garden J.O., Büchler M.W., Yokoe M. (2013). TG13 guidelines for diagnosis and severity grading of acute cholangitis (with videos). J. Hepatobiliary Pancreat. Sci..

[10] Peter A.B., Thomas L.B., Christos D., Hein G.G., Colin D.J., Michael G.S., Gregory G.T., Santhi S.V. (2013). Classification of acute Pancreatitis—2012: Revision of the Atlanta classification and definitions by international consensus. Gut..

[11] Sankar A., Johnson S.R., Beattie W.S., Tait G., Wijeysundera D.N. (2014). Reliability of the American Society of Anesthesiologists physical status scale in clinical practice. Br. J. Anaesth..

[12] Aronson W.L., McAuliffe M.S., Miller K. (2003). Variability in the American society of anesthesiologists physical status classification scale. AANA J..

[13] World Medical Association (2013). World Medical Association Declaration of Helsinki: Ethical principles for medical research involving human subjects. JAMA.

[14] Maple J.T., Ben-Menachem T., Anderson M.A., Appalaneni V., Banerjee S., Cash B.D., Fisher L., Harrison M.E., Fanelli R.D., Fukami N. (2010). The role of endoscopy in the evaluation of suspected choledocholithiasis. Gastrointest. Endosc..

[15] Sherman J.L., Shi E.W., Ranasinghe N.E., Sivasankaran M.T., Prigoff J.G., Divino C.M. (2015). Validation and improvement of a proposed scoring system to detect retained common bile duct stones in gallstone pancreatitis. Surgery.

[16] Yang M.H., Chen T.H., Wang S.E., Tsai Y.F., Su C.H., Wu C.W., Lui W.-Y., Shyr Y.M. (2008). Biochemical predictors for absence of common bile duct stones in patients undergoing laparoscopic cholecystectomy. Surg. Endosc..

[17] Salminen P., Laine S., Gullichsen R. (2008). Severe and fatal complications after ERCP: Analysis of 2555 procedures in a single experienced center. Surg. Endosc..

[18] Ying L., Tao Y., Qiang Y. (2018). Laparoscopic Ultrasonography Versus Magnetic Resonance Cholangiopancreatography in Laparoscopic Surgery for Symptomatic Cholelithiasis and Suspected Common Bile Duct Stones. J. Gastrointest. Surg..

[19] Anahita D., Abdul A., Sapan S.D., McMaster J., SreyRam K. (2014). National trends in the adoption of laparoscopic cholecystectomy over 7 years in the United States and impact of laparoscopic approaches stratified by age. Minim Invasive Surg..

[20] Peker Y., Ünalp H.R., Durak E., Karabuğa T., Yilmaz Y., Genç H., Haciyanli M. (2014). Laparoscopic cholecystectomy in patients aged 80 years and older: An analysis of 111 patients. Surg. Laparosc. Endosc. Percutan. Tech..

[21] Magalhaes J., Rosa B., Cotter J. (2015). Endoscopic retrograde cholangiopancreatography for suspected choledocholithiasis: From guidelines to clinical practice. World J. Gastrointest. Endosc..

[22] Czoski-Murray C., Lloyd J.M., McCabe C., Claxton K., Oluboyede Y., Roberts J., Nicholl J.P., Rees A., Reilly C.S., Young D. (2012). What is the value of routinely testing full blood count, electrolytes and urea, and pulmonary function tests before elective surgery in patients with no apparent clinical indication and in subgroups of patients with common comorbidities: A systematic review of the clinical and costeffective literature. Health Technol. Assess..

[23] Sharara A.I., Mansour N.M., El-Hakam M., Ghaith O., El Halabi M. (2010). Duration of pain is correlated with elevation in liver function tests in patients with symptomatic choledocholithiasis. Clin. Gastroenterol. Hepatol..

[24] Hallal A.H., Amortegui J.D., Jeroukhimov I.M., Casillas J., Schulman C.I., Manning R.J., Lopez P.P., Cohn S.M., Sleeman D., Habib F.A. (2015). Magnetic resonance cholangiopancreatography accurately detects common bile duct stones in resolving gallstone pancreatitis. J. Am. Coll. Surg..

[25] Taylor A.C., Little A.F., Hennessy O.F., Banting S.W., Smith P.J., Desmond P.V. (2002). Prospective assessment of magnetic resonance cholangiopancreatography for noninvasive imaging of the biliary tree. Gastrointest. Endosc..

[26] Topal B., Van de Moortel M., Fieuws S., Vanbeckevoort D., Van Steenbergen W., Aerts R., Penninckx F. (2003). The value of magnetic resonance cholangiopancreatography in predicting common bile duct stones in patients with gallstone disease. Br. J. Surg..

[27] Akisik M.F., Jennings S.G., Aisen A.M., Sherman S., Cote G.A., Sandrasegaran K., Tirke T. (2013). MRCP in patient care: A prospective survey of gastroenterologists. Roentgenology.

[28] Singhvi G., Ampara R., Baum J., Gumaste V. (2016). ASGE guidelines result in cost-saving in the management of choledocholithiasis. Ann. Gastroenterol..

[29] Adams M.A., Hosmer A.E., Wamsteker E.J., Anderson M.A., Elta G.H., Kubiliun N.M., Kwon R.S., Piraka C.R., Scheiman J.M., Waljee A.K. (2015). Predicting the likelihood of persistent bile duct stones in patients with suspected choledocholithiasis: Accuracy of existing guidelines and the impact of laboratory trends. Gatro.–intest. Endosc..

[30] Nárvaez Rivera R.M., González González J.A., Monreal Robles R., García Compean D., Paz Delgadillo J., Garza Galindo A.A., Maldonado-Garza H.J. (2016). Accuracy of ASGE criteria for the prediction of choledocholithiasis. Rev. Esp. Enferm. Dig..

[31] Sethi S., Wang F., Korson A.S., Krishnan S., Berzin T.M., Chuttani R., Pleskow D.K., Sawhney M.S. (2016). Prospective assessment of consensus criteria for evaluation of patients with suspected choledocholithiasis. Dig. Endosc..

[32] Verma D., Kapadia A., Eisen G.M., Adler D.G. (2006). EUS vs MRCP for detection of choledocholithiasis. Gastrointest. Endosc..

[33] Santambrogio R., Bianchi P., Opocher E., Verga M., Montorsi M. (1999). Prevalence and laparoscopic ultrasound patterns of choledocholithiasis and biliary sludge during cholecystectomy. Surg. Laparosc. Endosc. Percutan. Tech..

[34] Grier J.F., Cohen S.W., Grafton W.D., Gholson C.F. (1994). Acute suppurative cholangitis associated with choledochal sludge. Am. J. Gastroenterol..

[35] Mirbagheri S.A., Mohamadnejad M., Nasiri J., Vahid A.A., Ghadimi R., Malekzadeh R. (2005). Prospective evaluation of endoscopic ultrasonography in the diagnosis of biliary microlithiasis in patients with normal transabdominal ultrasonography. J. Gastrointest. Surg..

[36] Ryan M.J., Randy S.H., Ann M.R., Jerome R.L., Eric M.P. (2014). Antegrades Wire, Rendezvous Cannulation of the Biliary Tree May Reduce the Incidence of Post-ERCP Pancreatitis. Proceedings of the SAGES Abstract Archive.

[37] Francesco A.P., Mauro F., Marco B., Emy M., Antonella V., Bortolo P. (2015). Accuracy of magnetic resonance cholangiography compared to operative endoscopy in detecting biliary stones, a single center experience and review of literature. World J. Radiol..

[38] Tranter S.E., Thompson M.H. (2003). A prospective single-blinded controlled study comparing laparoscopic ultrasound of the common bile duct with operative cholangiography. Surg. Endosc..

[39] Perry K.A., Myers J.A., Deziel D.J. (2008). Laparoscopic ultrasound as the primary method for bile duct imaging during cholecystectomy. Surg. Endosc..

[40] Dili A., Bertrand C. (2017). Laparoscopic ultrasonography as an alternative to intraoperative cholangiography during laparoscopic cholecystectomy. World J. Gastroenterol..

